# 

**Published:** 1911-10

**Authors:** 


### Ye Editor's Outing.

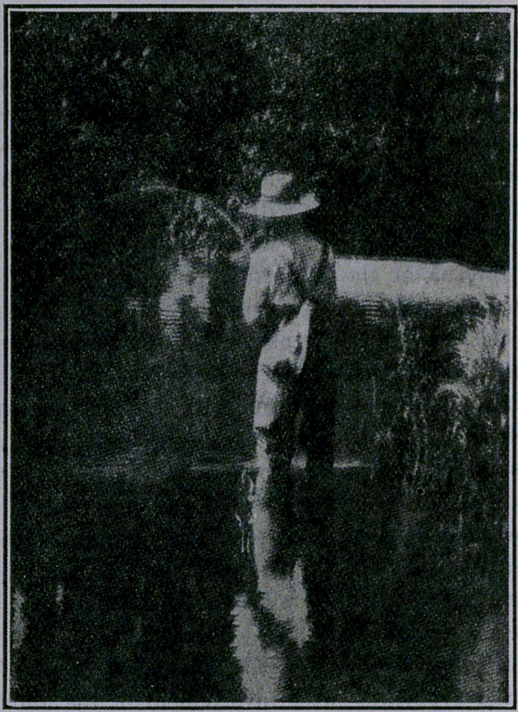

ON THE SAN MARCOS.

In a cool, sequestered nook where the clear and ice-cold waters of the San Marcos eddy and swirl beneath an overhanging rock, festooned with ferns and shaded by the cypress and the elm, there, with nice judgment, I cast the delusive lure. See the Monarch of the Stream dart out and seize it, with the swiftness of an arrow! Ha! The fight is on! Astonished and terror-stricken the big black bass—a four-pounder—darts hither and thither, dives down deep, tugging at the line, circles the pool once—twice—thrice, and leaps into the air, shaking his head like an angry bull. No use! The line holds—the reel sings—the finely tempered rod is true as steel, and Fate—with its relentless grasp—has snatched him from those haunts forever!

There is no thrill comparable to it; yet one feels a pang of regret to see the noble fellow lie gasping on the grass.

